# New Self-Repairing System for Brittle Matrix Composites Using Corrosion-Induced Intelligent Fiber

**DOI:** 10.3390/polym14183902

**Published:** 2022-09-18

**Authors:** Yuyan Sun, Dongkai Wang, Zuquan Jin, Jianwei Sun, Ziguo Wang

**Affiliations:** 1 School of Civil Engineering, Qingdao University of Technology, Qingdao 266520, China; 2 Engineering Research Center of Concrete Technology in Marine Environment, Ministry of Education, Qingdao University of Technology, Qingdao 266520, China

**Keywords:** brittle matrix composites, concrete, corrosion, crack, intelligent fiber, self-repair

## Abstract

Brittle matrix composites such as concrete are susceptible to damage in the form of cracks. Most of the current self-repair and self-healing techniques have repair limits on crack widths or high costs of an external stimulator, or have an unfavorable effect on the composite’s strength. This paper proposes a new concept of corrosion-induced intelligent fiber (CIF) and a new self-repairing system that uses the CIFs to close cracks in brittle matrix composites within a corrosive environment without external help, and without compromising the strength. The CIF comprises an inner core fiber and an outer corrodible coating that are in equilibrium, with the core fiber in tension and the corrodible coating in compression. The preparation steps and shape recovery mechanism of the CIF and the self-repair mechanism of the CIF composites are explained. Based on these concepts, this paper also describes several mechanical models built to predict the magnitude of pre-stress stored in the core fiber, and the maximum pre-stress released to the matrix composites, and the minimum length of the reliable anchor ends of CIF. The sample calculation results show that the recovery strain was 0.5% for the CIF with the steel core fiber and 12.7% for the CIF with the nylon core fiber; the maximum crack closing force provided by the CIF to concrete can be increased by increasing the amount of the CIFs in concrete and the initial tensile stress of the core fiber. This paper provides some suggestions for enhancing the self-repair capability of brittle composites in complex working environments.

## 1. Introduction

Brittle matrix composites such as ceramics, concrete, and brick are susceptible to cracking due to their low tensile strength, which can affect the overall mechanical performance and durability of the structures made of the composites [1,2,3]. Traditional repairing methods such as timed repair and after-the-fact repair can usually manage the visible cracks on the surface of some structural elements, but can hardly reach some cracks in complex structures. Self-repair or self-healing functionality of the composites, however, can provide timely repair to the structures and minimize the negative effect of cracks [3,4,5]—it is of great significance especially for the structures in complex environments such as space capsules, nuclear power plants, and marine tunnels.

Concrete is one of the most widely used construction materials. It is well known that concrete has some ability to self-heal [6]; its microdamage and microcracks caused by loads or environmental factors during the service time can be self-healed to some extent by further hydration of the unhydrated particles [7]. However, the self-healing efficiency of concrete seems to be low and the effect is limited by crack widths up to 200 μm [8,9,10]. To promote the self-repair or self-healing capacity of concrete, many techniques have been developed over the years, including: (1) Chemical self-healing techniques, such as mixing crystalline admixtures into concrete or brushing a layer of active admixture coating on the concrete surface [11,12,13,14,15,16,17], after water penetrates concrete through cracks, the active functional group undergoes a condensation reaction and produces CaCO_3_ crystals that fill the capillaries and microcracks in the concrete. When the concrete is again cracked, the active molecules are reactivated and continue to react until the cracks are healed. However, this method has minimal effect on repairing cracks over 400 μm in width [18,19,20]. (2) There are physical self-repair techniques, such as embedding shape memory alloy (SMA) into the crack-prone parts of concrete [21,22,23,24,25,26,27,28,29,30]; when cracks are generated, the electrical heating method is used to stimulate the shape recovery of SMA, forcing the cracks to close. However, the application scope of this technique is somewhat limited due to the relatively high investment in the external stimulation system and the expensive SMA. (3) Physical–chemical self-repair techniques, such as mixing the microencapsulated/hollow fibers (carriers) containing repair adhesive into concrete [31,32,33,34,35,36,37,38,39,40,41,42,43,44]—when cracks pass through the carrier, the repair agent flows out and penetrates into the cracks. The effect of this method is influenced by the number of carriers: fewer carriers are not enough to fill the cracks, while more carriers reduce the strength of the concrete. (4) Microbial self-healing techniques, such as adding specific harmless bacteria such as aerobic alkalophilic Bacillus into concrete [45,46,47,48,49,50,51,52,53,54,55,56]—as the concrete cracks, the infiltration of oxygen and water activates the dormant bacterial spores, and the process of microbial metabolism produces CO_2_ that reacts with Ca^2+^ in the concrete to produce CaCO_3_ crystals that seal and repair cracks up to 500 μm wide [57,58,59]; however, this method has some environmental and practical limitations because the bacteria have certain requirements concerning working environment and temperature. (5) Electrochemical self-repair techniques, such as the electrochemical deposition method, are generally achieved in an electric field where the positive and negative ions in the solution are deposited in the crack through an electrode reaction, producing insoluble crystals that fill the cracks [60,61,62,63,64,65,66,67,68,69,70]. However, this method requires the entire repair process to be electrified and the cost is relatively high.

As mentioned above, most of the current self-repair and self-healing techniques have repair limitations on crack width, or have an unfavorable effect on concrete strength, or need extra investment in an external stimulus. In contrast, the addition of fibers into concrete, though they cannot close or repair cracks, is helpful to control and reduce cracking and usually results in improved concrete strength and toughness, so it often combines with some self-repair or self-healing technique to achieve crack closure [3,71,72,73,74,75,76]. However, for the structures in corrosive environmental conditions, once the concrete is cracked, the harmful substances can enter through the cracks and rapidly corrode the reinforcement and cause overall degradation of structural integrity; in this case, the effect of the current self-repair or self-healing methods is even more limited. Based on these issues, we hope to develop a novel and relatively economical self-repair system that can enhance the durability of concrete structures under harsh environmental conditions.

This paper proposes a new concept of corrosion-induced intelligent fiber (CIF) and a new self-repairing system that uses the CIFs to close cracks in the brittle matrix composites within a corrosive environment without external help. The CIF can be prepared by coating the surface of the pre-tensioned core fiber with a corrosion-prone coating, then the CIFs can be embedded in the crack-prone parts of the brittle matrix composites. Once cracking occurs, the corrosive media in the environment penetrate through the cracks and trigger the shrinkage of the CIFs, which in turn releases the stored pre-stress to drive the cracks in the brittle matrix composites to close. Based on this concept, this paper also builds several mechanical models of the CIF and the self-repairing system to predict the magnitude of pre-stress stored in the core fiber, and the maximum pre-stress released to the brittle matrix composite, and the minimum length of the reliable anchor ends of CIF. This paper aims to provide the conceptual and theoretical development of the new CIF that can be efficient for closing cracks with a wide width range without external help, and without compromising strength, which has the potential to realize the intelligent functions of the brittle matrix composites in their feedback to external corrosion damage.

## 2. Corrosion-Induced Intelligent Fiber (CIF)

### 2.1. General Concept

**Figure 1:**
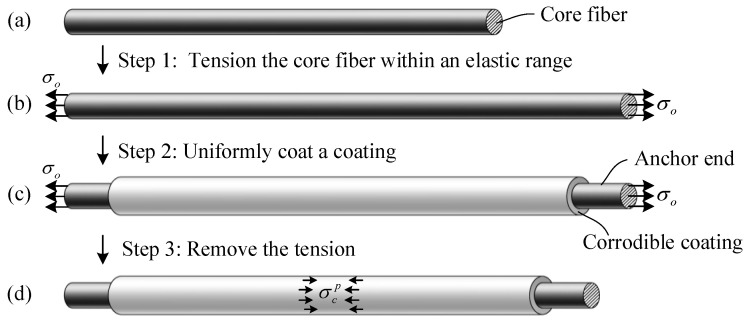
Schematic diagram of preparation process of the corrosion-induced intelligent fiber (CIF), (**a**) unstressed core fiber, (**b**) pre-tensioned core fiber, (**c**) pre-tensioned core fiber coated with an unstressed corrodible coating, (**d**) CIF in equilibrium with core fiber in tension and corrodible coating in compression.

The new CIF comprises an inner core fiber and an outer corrodible coating, wherein the core fiber is a corrosion-resistant material, and the corrodible coating can be easily corroded by corrosive media in the environment. The preparation steps of the CIF are shown in Figure 1. First, the core fiber is in an unstressed state (see Figure 1a), secondly, it is pre-tensioned in an elastic range, and the tensile stress is *σ*_0_ (see Figure 1b), thirdly, the surface of the core fiber is uniformly coated with a corrodible coating by deposition, spraying, electroplating, or a similar process when the tensile stress *σ*_0_ remains constant and the corrodible coating is in an unstressed state (see Figure 1c), and fourthly, the tensile force is removed after the coating is completed and cured. It is assumed that the core fiber is well bonded to the corrodible coating. In the process of removal, the corrodible coating is axially compressed under the elastic recovery force of the core fiber, and the compressive stress is $\sigma_{c}^{p}$ (see Figure 1d). Finally, a tensile–compressive equilibrium is established between the corrodible coating and the core fiber, with the former in compression and the latter in tension.

### 2.2. Shape Recovery Mechanism of CIF

**Figure 2:**
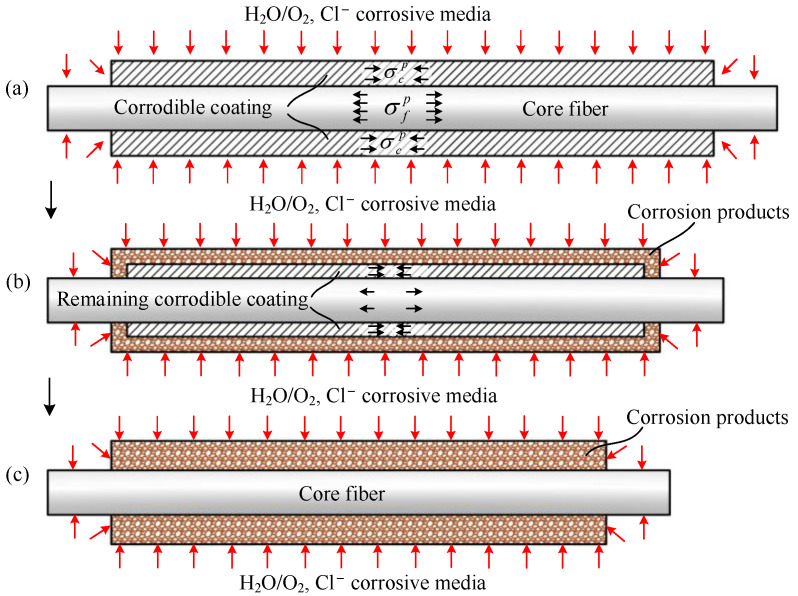
Axial cross-sectional view of the shape recovery mechanism of CIF, (**a**) CIF is in contact with corrosive media, (**b**) the effective force section of the corrodible coating decreases, the equilibrium is broken, and the core fiber shrinks, (**c**) core fiber recovers to the original length after the corrodible coating being corroded thoroughly.

The shape recovery mechanism of the CIF is shown in Figure 2. When the environment is not corrosive, the CIF is not corroded, and the core fiber and the corrodible coating are in an original equilibrium state. In the corrosive environment, the corrodible coating in contact with the corrosive media forms a load-unbearable corrosion product, so the effective force section of the corrodible coating is decreasing, and the equilibrium state is broken; thus, the compressive stress and compressive deformation of the remaining corrodible coating increase constantly under the elastic recovery force of the core fiber, thereby the core fiber shrinks and gradually approaches its initial length, as shown in Figure 2b. Figure 2c shows that after the corrodible coating is corroded thoroughly, the pre-stress in the CIF is released and the core fiber recovers to the original length in an unstressed state.

## 3. Self-Repair Principle of CIF Composites

**Figure 3:**
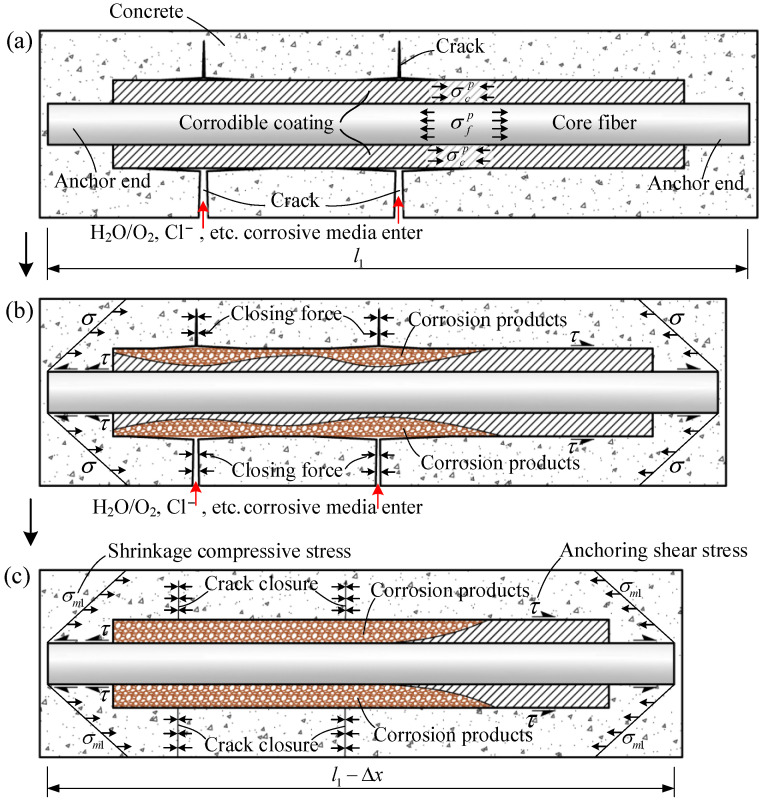
Axial cross-sectional view of the self-repair principle of the CIF composite, (**a**) corrosive media enter along the cracks and react with the corrodible coating, (**b**) CIF is triggered to shrink, the pre-stress stored in the core fiber is released to the matrix composite, (**c**) after the corrodible coating is corroded to a certain extent, the pressure applied to the matrix composite is large enough to close the cracks.

The CIF can be embedded in the crack-prone parts of the brittle matrix composite when applied in a corrosive environment. In order to transfer the pre-stress to the matrix composites more effectively, preferably the CIF reserve reliable anchor ends, such as uncoated bare ends, gradually thickening ends or end hooks. In the presence of the reliable anchor ends, whether the crack is distributed at the end portion of the CIF or the corrodible coating is completely corroded, the fiber is unlikely to be pulled out. The principle of self-repair of the CIF composites is shown in Figure 3. When the matrix composite cracks and the crack tips develop to the corrodible coating of the CIF, the corrosive media enter along the cracks and chemically or electrochemically react with the corrodible coating, and the CIF is triggered to shrink and transfers load through the bonding interface between the uncorroded CIF and the matrix composite to apply pressure to the matrix composite (see Figure 3b). It is clear that the higher corrosion degree of the corrodible coating, the larger crack closing force and a smaller crack width is generated. After the corrodible coating is corroded to a certain extent, the crack closing force is large enough, and the cracks are closed, as shown in Figure 3c; therefore, the inner passage for the corrosive media is cut off, and the corrosion is stopped, so the self-repair function is realized. At this time, the shrinkage of the CIF stops without increasing pressure to the brittle matrix composite.

## 4. Derivation of the Mechanical Model

### 4.1. Mechanical Model of CIF

#### 4.1.1. Basic Assumption

Since the CIF is a unidirectional composite with a large enough slenderness ratio, in order to simplify the calculation of internal force of the CIF, the following assumptions may be made:

1. The corrodible coating is evenly coated on the core fiber;
2. The core fiber and the corrodible coating are well bonded at the interface and the two have good chemical compatibility;
3. The influence of transverse strain of the core fiber and the corrodible coating is ignored and not incorporated into the Poisson’s ratio in formula derivation;
4. The force of the core fiber and the corrodible coating is in a linear elastic state;
5. The structural unit is pulled positive and compressed negative.

#### 4.1.2. Calculation of Internal Force

The symbols used in this section are listed in Table A1.

**Figure 4:**
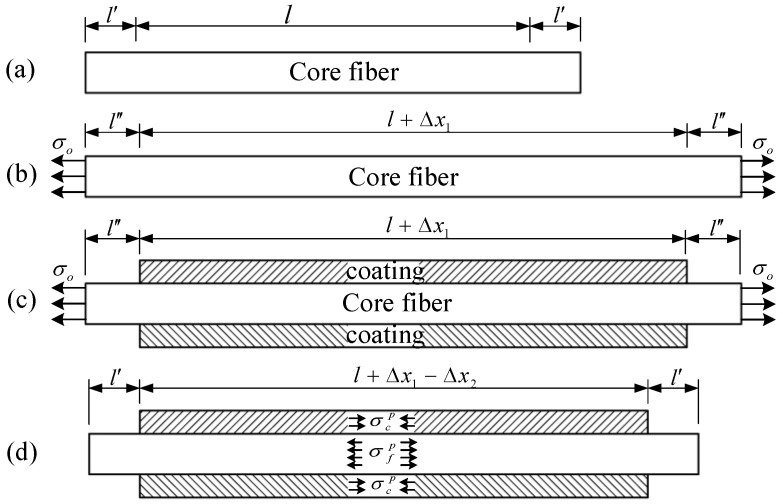
The process diagram of CIF force equilibrium, (**a**) unstressed stage, (**b**) pre-tension stage, (**c**) coating stage, (**d**) external force withdrawal stage.

As shown in Figure 4, it is assumed that the original length of the core fiber that will be coated by the corrodible coating is *l* (see Figure 4a); in the pre-tensioning stage (see Figure 4b), the tensile stress is *σ*_0_, and the elongation of the core fiber is ∆*x*_1_; in the coating stage (see Figure 4c), the length of the deposited coating is *l* + ∆*x*_1_. Owing to the recovery force of the core fiber after removing the pre-tension (see Figure 4d), the compressive deformation of the coating is ∆*x*_2_, and the core fiber and the coating achieve force equilibrium and coordinated deformation. The tensile force of the core fiber is obtained according to Hooke’s law as

(1) $$F_{f}=\frac{E_{f}A_{f}}{l}\left(\Delta x_{1}-\Delta x_{2}\right)$$

The pressure of the corrodible coating is

(2) $$F_{c}=\frac{E_{c}A_{c}}{l+\Delta x_{1}}\left(-\Delta x_{2}\right)$$

The force equilibrium requires *F_f_* + *F_c_* = 0, that is

(3) $$\frac{E_{f}A_{f}}{l}\left(\Delta x_{1}-\Delta x_{2}\right)+\frac{E_{c}A_{c}}{l+\Delta x_{1}}\left(-\Delta x_{2}\right)=0$$

then

(4) $$\Delta x_{2}=\frac{E_{f}A_{f}\Delta x_{1}}{\frac{E_{c}A_{c}l}{l+\Delta x_{1}}+E_{f}A_{f}}$$

Since the compressive stress in the corrodible coating is

(5) $$\sigma_{c}^{p}=E_{c}\varepsilon_{c}=E_{c}\frac{-\Delta x_{2}}{l+\Delta x_{1}}$$

Substituting Equation (4) into Equation (5) gives

(6) $$\sigma_{c}^{p}=-\frac{E_{c}E_{f}A_{f}\Delta x_{1}}{E_{c}A_{c}l+E_{f}A_{f}\left(l+\Delta x_{1}\right)}$$

Supposing the cross-sectional area of the CIF is *A* = *A_c_* + *A_f_*, and simultaneously dividing the numerator and denominator on the right side of Equation (6) by *Al*, then

(7) $$\sigma_{c}^{p}=-\frac{E_{c}E_{f}V_{f}\varepsilon_{f}}{E_{c}V_{c}+E_{f}V_{f}\left(1+\varepsilon_{f}\right)}$$

where *ε_f_* = Δ*x*_1_/*l*; substituting *ε_f_* = *σ*_0_/*E_f_* into Equation (7), then

(8) $$\sigma_{c}^{p}=-\frac{E_{c}V_{f}\sigma_{o}}{E_{c}V_{c}+E_{f}V_{f}+V_{f}\sigma_{o}}$$

As *σ*_0_ is much smaller than *E_f_*, thus,

(9) $$\sigma_{c}^{p}\approx -\frac{E_{c}V_{f}\sigma_{o}}{E_{c}V_{c}+E_{f}V_{f}}$$

At this point, the expression of pre-stress stored in the core fiber is

(10) $$\sigma_{f}^{p}=-\frac{\sigma_{c}^{p}V_{c}}{V_{f}}=\frac{E_{c}V_{c}\sigma_{o}}{E_{c}V_{c}+E_{f}V_{f}}=\frac{E_{c}V_{c}\sigma_{o}}{E_{1}}$$

where *E*_1_ = *E_c_V_c_* + *E_f_V_f_* is the composite elastic modulus, and *V_c_* + *V_f_* = 1.

#### 4.1.3. Force Storage Optimization

Based on Equation (10), the axial force *F* stored in the core fiber is

(11) $$F=\sigma_{f}^{p}A_{f}=\frac{E_{c}V_{c}\sigma_{o}A_{f}}{E_{c}V_{c}+E_{f}V_{f}}=\frac{E_{c}V_{c}\sigma_{o}V_{f}A}{E_{c}V_{c}+E_{f}V_{f}}=\frac{\left(1-V_{f}\right)V_{f}}{E_{c}\left(1-V_{f}\right)+E_{f}V_{f}}E_{c}\sigma_{o}A$$

When *F* is maximum, the pre-stress released to the matrix composite is maximum. To solve the maximum value of the axial force of the core fiber, the *F* is first derived to obtain

(12) $$F^{\prime }=\frac{\left(1-2V_{f}\right)\left[E_{c}\left(1-V_{f}\right)+E_{f}V_{f}\right]-\left(V_{f}-V_{f}^{2}\right)\left(E_{f}-E_{c}\right)}{{\left[E_{c}\left(1-V_{f}\right)+E_{f}V_{f}\right]}^{2}}E_{c}\sigma_{o}A$$

that is

(13) $$F^{\prime }=\frac{\left(E_{c}-E_{f}\right)V_{f}^{2}-2E_{c}V_{f}+E_{c}}{{\left[E_{c}\left(1-V_{f}\right)+E_{f}V_{f}\right]}^{2}}E_{c}\sigma_{o}A$$

When *F*’ = 0, then

(14) $$\left(E_{c}-E_{f}\right)V_{f}^{2}-2E_{c}V_{f}+E_{c}=0$$

When *E_c_* = *E_f_*, the *F* is maximum, and *V_f_* = 0.5; when *E_c_* ≠ *E_f_*, for the equation

(15) $$V_{f}^{2}-\frac{2E_{c}}{E_{c}-E_{f}}V_{f}+\frac{E_{c}}{E_{c}-E_{f}}=0$$

Assuming $a=\frac{E_{C}}{E_{C}-E_{f}}$, since *E_c_* > 0 and *E_f_* > 0, then *a* < 0 or *a* > 1, thus Δ = 4*a*^2^ – 4*a* > 0 and Equation (15) has two different real roots, which are

(16) $$V_{f}=a\pm \sqrt{a^{2}-a}=\frac{E_{c}\pm \sqrt{E_{c}E_{f}}}{E_{c}-E_{f}}=\frac{1\pm \sqrt{E_{f}/E_{c}}}{1-E_{f}/E_{c}}$$

Since the real root $V_{f}=\frac{1+\sqrt{E_{f}/E_{C}}}{1-E_{f}/E_{C}}$ does not satisfy the condition 0 < *V_f_* < 1, it should be discarded, while the other real root

(17) $$V_{f}=\frac{E_{c}-\sqrt{E_{c}E_{f}}}{E_{c}-E_{f}}$$

satisfies the condition and gives the maximum axial force storage *F_max_*.

### 4.2. Mechanical Model of CIF Composites

The permanent anchor ends of CIF are the portion of the core fiber not coated with the corrodible coating, or the portion where the surface of the core fiber with corrodible coating is coated with the corrosion-resistant coating; the length of any permanent anchor end is defined as *l’*. When the CIF reserved with permanent anchor ends is added into the matrix composite, the pre-stress released to the matrix composite can be predicted when the shrinkage of CIF stops.

#### 4.2.1. Basic Assumption

In order to simplify the calculation of interaction between the CIF and the brittle matrix composite, the following assumptions are made:

1. The CIF is unidirectionally and uniformly arranged in the matrix composite;
2. The influence of the Poisson’s ratio on the magnitude of the axial stress is disregarded;
3. The permanent anchor ends are tightly bonded with the matrix composite without slippage;
4. The force influence of the corrosion product of the corrodible coating is disregarded.

#### 4.2.2. Calculation of Internal Force

The symbols used in this section are listed in Table A2.

After the cross-section of the corrodible coating is completely lost, the pre-stress released to the brittle matrix composite by the shrinkage of the core fiber is maximum. As the corrosion product does not participate in the force, the core fiber and the brittle matrix composite establish a final tensile–compressive equilibrium. According to Equation (9), it can be known that the pre-stress released to the matrix composite by the shrinkage of the core fiber is

(18) $$\sigma_{m}^{p}=-\frac{E_{m}\frac{V_{f1}}{1-V_{c1}}\sigma_{f}^{p}}{E_{2}}=-\frac{E_{m}V_{f1}\sigma_{f}^{p}}{E_{f}V_{f1}+E_{m}V_{m}}$$

At this point, the tensile stress in the core fiber is

(19) $$\sigma_{f1}^{p}=\frac{E_{m}V_{m}\sigma_{f}^{p}}{E_{f}V_{f1}+E_{m}V_{m}}$$

where *E*_2_ is the composite elastic modulus of the brittle matrix composite with the core fiber, and *E*_2_ = *E_f_V_f_*_1_/(1 – *V_c_*_1_) + *E_m_V_m_*/(1 – *V_c_*_1_), *V_f_*_1_ + *V_c_*_1_ = *V_s_*, and *V_s_* + *V_m_* = 1.

#### 4.2.3. Anchor Length of CIF

In order that the permanent anchor ends are reliable without slipping, a sufficient length is required. It is assumed that the bonding anchoring force of the permanent anchor end is *T_a_* = *τπdl*′, and the drawing force of the CIF is $T_{t}=\sigma_{f1}^{p}\pi d^{2}/4$. According to the force equilibrium *T_a_* = *T_t_*, the following is obtained

(20) $$l^{\prime }=\frac{d\sigma_{f1}^{p}}{4\tau }$$

where *τ* is the bonding stress between the CIF and the matrix composite at the interface, and when the composition and properties of the matrix composite and the CIF are known, *τ* can be determined; *l’* is the anchor length (the length of one end) of the CIF in the brittle matrix composite, and *d* is the diameter of the cross-section of the anchor end.

Formula (19) is substituted into Equation (20) to obtain

(21) $$l^{\prime }=\frac{d\sigma_{f1}^{p}}{4\tau }=\frac{dE_{m}V_{m}\sigma_{f}^{p}}{4\tau \left(E_{f}V_{f1}+E_{m}V_{m}\right)}$$

If the permanent anchor end is reliable for effectively transferring the pre-stress to the matrix composite without slipping, then

(22) $$l^{\prime }\geq \frac{dE_{m}V_{m}\sigma_{f}^{p}}{4\tau \left(E_{f}V_{f1}+E_{m}V_{m}\right)}$$

Thus, for the given material parameters of the CIF and the matrix composite, and the given volume fraction, the minimum length of the reliable anchor end of the CIF can be confirmed by calculation.

## 5. Discussion

Based on the concept of CIF, the self-repair method of the CIF composites has obvious advantages. First, the CIF composites working in a corrosive environment are capable of self-repairing without external help and independent of temperature, so compared to the self-repair techniques using SMA or an electric field, the use of CIF in concrete can reduce the costs. Second, the larger the pre-stress stored in the core fiber, or the higher corrosion degree the corrodible coating encounters, the larger the crack closure force that can be released to the concrete, meaning a wider crack can be repaired; thus, compared to the self-healing or self-repair techniques using crystalline admixtures, microcapsules, or bacteria, the use of CIF in concrete can close cracks with a wider width range without compromising the strength of the concrete. Third, before the corrodible coating is corroded thoroughly, when the concrete is again cracked, the corrodible coating can continue to be corroded until the cracks are closed or the cross-section of the corrodible coating is completely lost. Fourth, the use of CIF in concrete can act as effective reinforcement both before and after corrosion.

Based on the derived mechanical models, we can predict the self-repair capacity of the CIF composite. For example, we set the material of the corrodible coating of the CIF to be iron, the core fiber of the CIF to be a copper-plated steel fiber or a nylon fiber with a diameter of 0.2 mm (regardless of copper plating amount), and set the matrix composite to be concrete in a chloride environment. The material parameters of CIF and concrete are listed in Table 1. According to Section 4.1.3, if *E_c_* = *E_f_*, the maximum axial force stored in the core fiber (*F_max_*) is obtained when the volume fraction of the core fiber in the CIF was 50%; if *E_c_* ≠ *E_f_*, the *F_max_* is obtained when the volume fraction of the core fiber is determined according to Equation (17). Setting the amount of CIFs in concrete (*V_s_*) to be 4V%, and assuming that the CIF is unidirectionally and uniformly arranged in the concrete, then the pre-stress stored in the core fiber ($\sigma_{f}^{p}$) and the maximum pre-stress released to the concrete ($\sigma_{m}^{p}$) are calculated according to Equations (10) and (18), respectively. The recoverable strain of the core fiber ($\varepsilon_{f}^{p}$) can be calculated according to $\varepsilon_{f}^{p}=\sigma_{f}^{p}/E_{f}$. The results are shown in Table 1.

**Figure 5:**
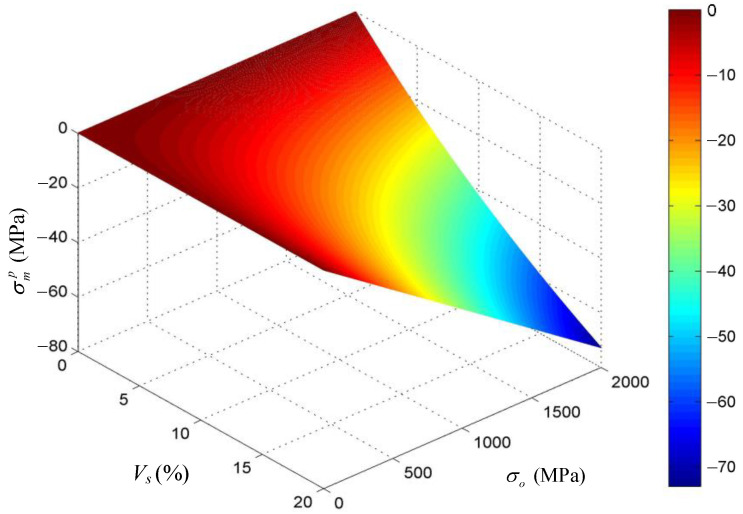
The influence of the amount of CIFs (*V_s_*) and the initial tensile stress ($\sigma_{0}$) on the maximum pre-stress released to concrete ($\sigma_{m}^{p}$ ).

From the above sample calculation, it can be shown that, after the iron coating of the CIF is lost due to chloride corrosion, the recovery strain of the core fiber is 0.5% for the steel fiber and 12.7% for the nylon fiber. That is, if the length of the CIF is 20 mm, the recovery strain can be 2.5 mm for the CIF with the nylon core fiber, which means, if the CIF concrete member is free of external force, the theoretical crack closure width can be up to 2.5 mm. It can also be seen from Table 1 that the maximum pre-stress released to the concrete is 18.6 MPa for the CIF with the steel core fiber and 24.5 MPa for the CIF with the nylon core fiber. Figure 5 shows that when the amount of CIFs (*V_s_*) and the initial tensile stress of the core fiber ($\sigma_{0}$) continuously increase, the maximum compressive stress applied to concrete also continuously increases. Thus, for the given composition and properties of the concrete and the CIF, the crack closing force provided by the CIF to concrete can be increased by increasing *V_s_* and $\sigma_{0}$.

The application of CIF will benefit crack closure, increase rigidity, and improve the corrosion resistance of concrete. This paper only provides the conceptual and theoretical study of the CIF and CIF composites; many important issues remain for future study. First, the performance of the self-repairing system and the optimization of the composition and properties of the CIF need to be explained via finite element analysis. Second, the optimized CIF needs to be prepared in the laboratory and explored with a set of experiments that can show the self-repairing behavior of the CIF composite within a corrosive environment.

## 6. Conclusions

This paper presents a new concept of CIF that comprises an inner corrosion-resistant core fiber and an outer corrodible coating that can be easily corroded by corrosive media in the environment. During preparation process, the inner core fiber is put into tension and the outer corrodible coating into compression, such that the CIF is in equilibrium. When the CIF is in contact with corrosive media, the outer corrodible coating is corroded, and the core fiber shrinks and displays shape recovery, which in turn releases the pre-tension stress in the core fiber. By far, shape memory fibers comprising core fibers coated with a corrosion-resistant compound/material are well known, but a shape memory fiber by coating a corrodible coating on a core fiber has not yet been reported.

This paper also proposes a new self-repairing system that uses the CIFs to close cracks in brittle matrix composites within a corrosive environment. Once cracking occurs, the CIFs embedded in the matrix composite can be triggered to shrink by the corrosive media from the environment, which in turn releases the pre-stress stored in the core fiber and thereby applies a compressive force to the matrix composite that acts to close the cracks. By far, self-repair concrete comprising reinforcing fibers is well known, but not with a corrodible coating in equilibrium with the core fiber. Compared to the current self-repair or self-healing techniques for concrete, the use of CIF in concrete can cost less than using SMA or the electrochemical deposition method because it is independent of temperature and does not need external help. Furthermore, it can be more efficient for closing wider cracks than that provided by the crystalline admixtures, microcapsules, or bacteria methods, all of which have unfavorable effect on concrete strength. Additionally, the use of CIF in brittle matrix composite can act as effective reinforcement both before and after corrosion.

Based on the concepts, this paper also builds several mechanical models to predict the magnitude of pre-stress stored in the core fiber, the maximum pre-stress released to the brittle matrix composite, and the minimum length of the reliable anchor ends of CIF. These aim to attain an optimum combination of the CIF and matrix composite to provide enough crack closing force. Based on a sample calculation, the recovery strain was 0.5% for CIF with a steel core fiber and 12.7% for CIF with a nylon core fiber. The maximum crack closing force provided by the CIF to concrete can be increased by increasing the amount of CIFs in concrete and the initial tensile stress of the core fiber.

The presence of CIF can be helpful toward improving the crack resistance of concrete, especially the low-modulus polymer fiber concrete. It can help to reduce the probability of premature concrete cracking and improve the durability of the concrete structures in corrosive environments, including marine and underground environments. For the future work, many important issues related to the concepts need to be explored. First, the optimization of the composition and properties of the CIF needs to be found via finite element analysis before performing the time-consuming laboratory tests; second, the optimized CIF needs to be prepared in the laboratory; and third, a set of experiments should be conducted to explore the self-repairing behavior of the CIF composite within actual corrosive environments.

## Figures and Tables

**Table 1:** Basic material parameters and calculation results of CIF and concrete.

No.	Composition	Material	Parameters			$\sigma_{f}^{p}$ (MPa)	$\varepsilon_{f}^{p}$	$\sigma_{m}^{p}$ (MPa)
			*E* (GPa)	*V_f_*_1_	*σ*_o_ (MPa)			
1	Corrodible coating	Iron	200	50% × 4%	-	-	-	-
	Core fiber	Steel	200	50% × 4%	2000	1000	0.5%	−18.6
	Matrix	Concrete	35	96%	-	-	-	-
2	Corrodible coating	Iron	200	14% × 4%	-	-	-	-
	Core fiber	Nylon	5.4 [77]	86% × 4%	800	687	12.7%	−24.5
	Matrix	Concrete	35	96%	-	-	-	-

## Data Availability

No new data were created or analyzed in this study. Data sharing is not applicable to this article.

## Appendix A

**Table A1:** Symbols in formula deduction for the corrosion-induced intelligent fiber (CIF).

Symbol	Description
*E_f_*	elastic modulus of the core fiber
*E_c_*	elastic modulus of the corrodible coating
*A_f_*	cross-sectional area of the core fiber
*A_c_*	cross-sectional area of the corrodible coating
$\varepsilon_{f}$	initial tensile strain of the core fiber
$\varepsilon_{c}$	strain of the corrodible coating after equilibrium
*V_f_*	volume fraction of the core fiber in the CIF
*V_c_*	volume fraction of the corrodible coating in the CIF
*E*_1_	elastic modulus of the CIF
$\sigma_{0}$	pre-tensile stress in the core fiber
$\sigma_{f}^{p}$	pre-stress stored in the core fiber after equilibrium
$\sigma_{c}^{p}$	compressive stress in the corrodible coating after equilibrium

**Table A2:** Symbols in formula deduction for CIF composites.

Symbol	Description
*E_m_*	elastic modulus of the brittle matrix composite
*E*_2_	composite elastic modulus of the matrix composite with the core fiber
*V_f_*_1_	volume fraction of the core fiber in the CIF composite
*V_c_*_1_	volume fraction of the corrodible coating in the CIF composite
*V_s_*	volume fraction of the CIFs in the CIF composite
*V_m_*	volume fraction of the matrix composite in the CIF composite
$\sigma_{m}^{p}$	pre-stress released to the brittle matrix composite after equilibrium
$\sigma_{f1}^{p}$	tensile stress in the core fiber after equilibrium
